# Supplementary figures and images for: Identifying app components that promote physical activity: a group concept mapping study
Source: PeerJ. 2024 Mar 29;12:e17100. doi: 10.7717/peerj.17100 (PMC10984184; doi:10.7717/peerj.17100)

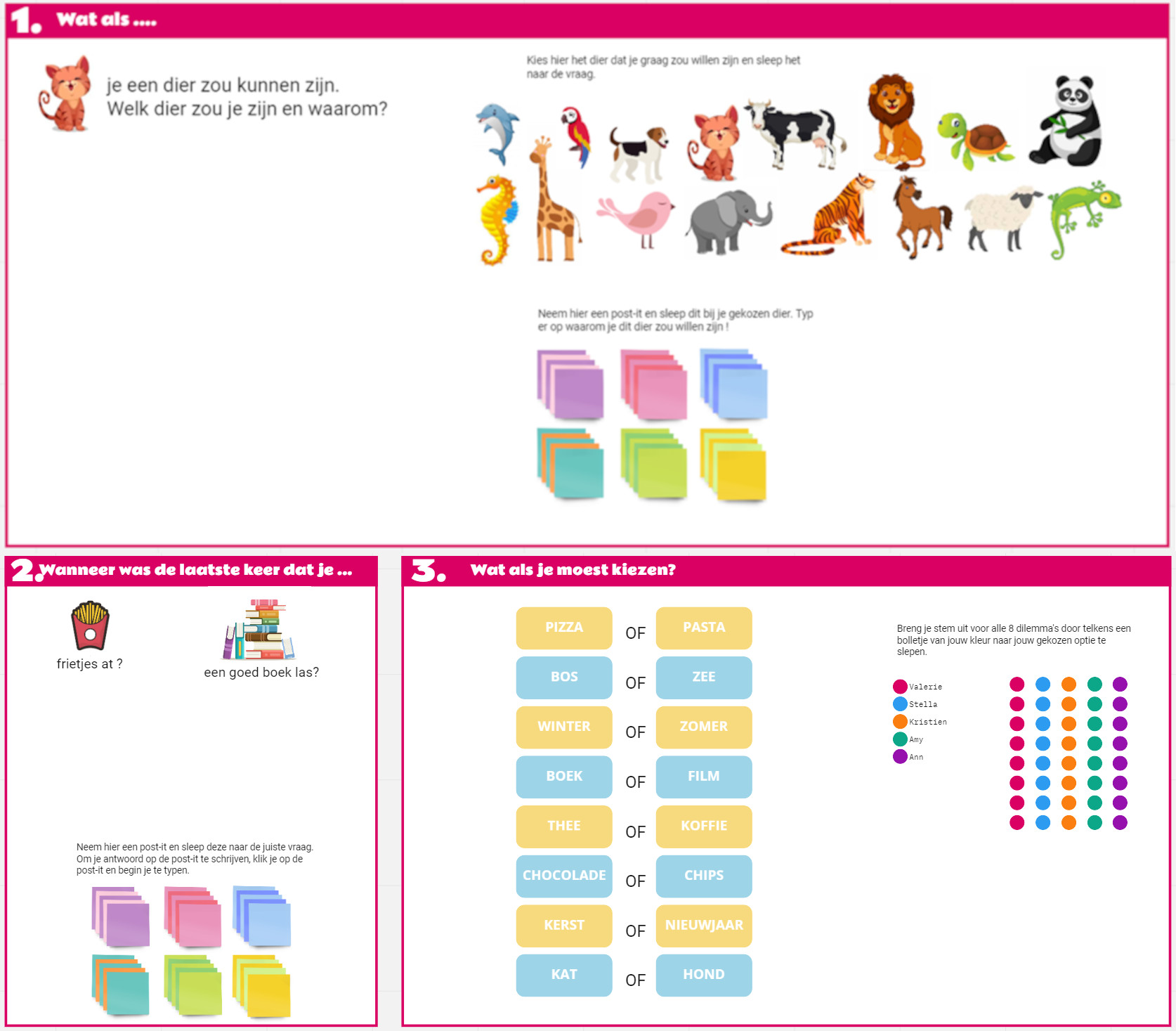

Supplement: Supplemental Information 1 [file peerj-12-17100-s001.jpg]

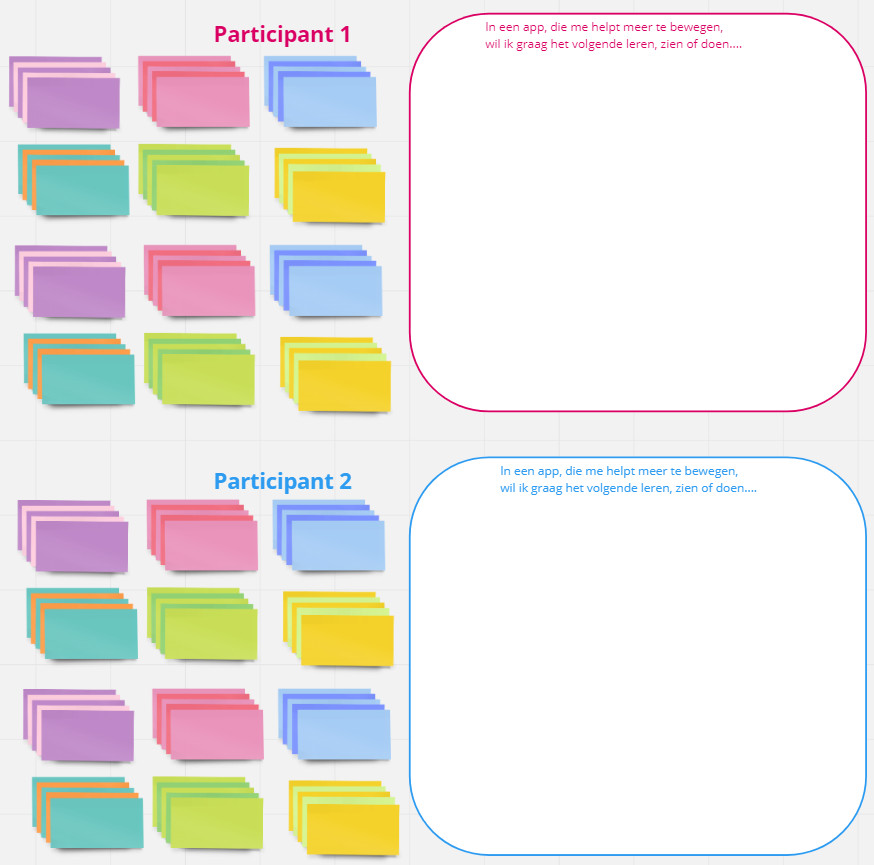

Supplement: Supplemental Information 2 [file peerj-12-17100-s002.jpg]

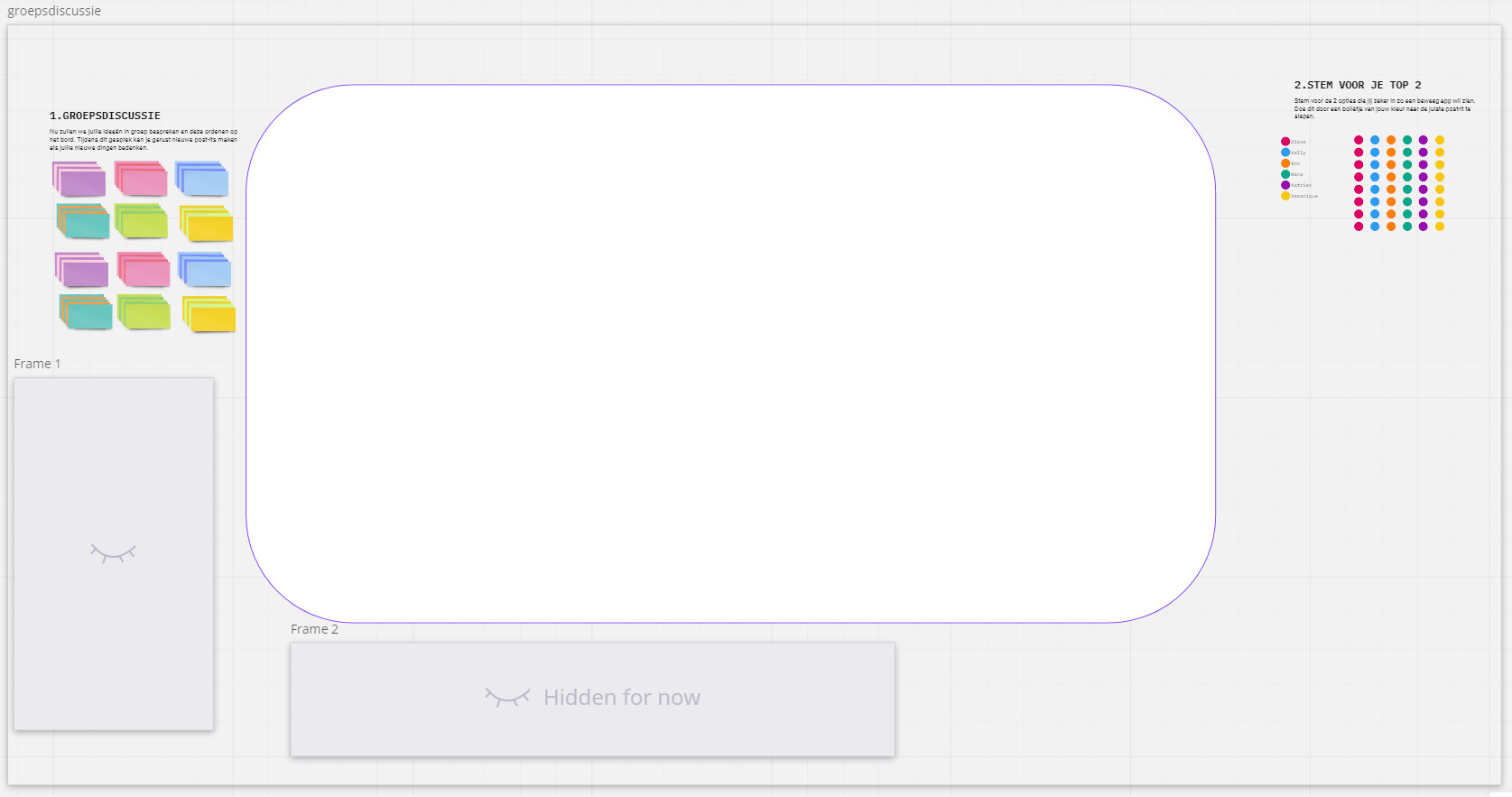

Supplement: Supplemental Information 3 [file peerj-12-17100-s003.jpg]
